# Caregivers’ absence from work before and after tonsil surgery in children with sleep-disordered breathing

**DOI:** 10.1007/s00405-020-06243-w

**Published:** 2020-08-07

**Authors:** Johanna Gudmundsdottir, Josefine Lindgren, Malin Thorpö, Helena Johansson, Johan Hellgren, Gunnhildur Gudnadottir

**Affiliations:** 1 Department of Otorhinolaryngology, Head and Neck Surgery, Institute of Clinical Sciences, Sahlgrenska University Hospital, University of Gothenburg, Gröna Stråket 9, 413 45 Göteborg, Sweden; 2 Mary McKillop Institute for Health Research, Australian Catholic University, Melbourne, Australia

**Keywords:** Sleep-disordered breathing, Children, Temporary parental benefit, Tonsillectomy, Tonsillotomy

## Abstract

**Purpose:**

Sleep-disordered breathing, SDB, in children is associated with morbidity that can result in caregivers having to stay at home from work. The aim of this study was to investigate whether the number of days when caregivers are reimbursed, temporary parental benefit (TPB) to stay at home from work to care for their sick child is increased among children with SDB before and after tonsil surgery.

**Methods:**

This is a retrospective, cross-sectional study of children (*n* = 440), aged 2–11 years, that underwent tonsil surgery for SDB in day surgery at Sahlgrenska University Hospital in 2014 and 2015. TPB, was provided by the Swedish Social Insurance Agency. The expected days of TPB in the general population of the region were calculated. The number of days with TPB was compared 2 years before and 2 years after surgery and compared with the expected days of TPB.

**Results:**

Two years before surgery, the children had no more days of TPB than expected. Two years after surgery, the children with SDB had 4.8 more days with TPB (*p* < 0.001) than expected, but, when the 1st month after surgery was excluded from the analysis, there was no difference in days of TPB compared with the general population.

**Conclusion:**

Children with SDB who had tonsil surgery had no more days of parental benefits 2 years before and 2 years after surgery than expected. SDB is associated with increased morbidity, but it does not appear to cause caregivers to stay at home in the majority of children.

## Introduction

Sleep-disordered breathing (SDB) is a common disease in children. The prevalence is estimated at 4–11%, based on parent-reported symptoms [1]. SDB is associated with multiple health problems and impaired quality of life. Sleep fragmentation and hypoxaemia can cause systemic inflammation and neurocognitive and behavioural problems [2–5], as well as growth retardation, and negative effects on the cardiovascular system [6]. An association with asthma and allergic rhinitis has also been reported [7]. Tarasiuk et al. found an increase in general healthcare utilisation among children in the years prior to their SDB diagnosis that was predominantly linked to more upper respiratory infections [8]. This morbidity and associated healthcare costs decreased after treatment [9].

The most common cause of SDB in children is upper airway obstruction due to adenotonsillar hypertrophy [10]. Both surgery and medical treatment with intranasal steroids have been shown to be effective in children with SDB, whereas surgery on the adenoid and tonsils is the predominant treatment modality [11, 12]. Children with SDB who undergo tonsil surgery improve in terms of behaviour, quality of life and polysomnographic findings [11].

One important aspect of the negative health effects associated with SDB and its treatment is whether or not the parents have to stay at home from work to take care of their sick child and whether that need is reduced after treatment. To our knowledge, this has not been studied before. Having to stay home to take care of a sick child imposes health-economic costs on the individual and on society at large, due to lost production and reimbursement provided to the caregiver. It could also be an indication of the severity of SDB-associated morbidity. Having to stay at home from work is, therefore, likely to add to the overall burden of paediatric SDB.

In Sweden, a caregiver is entitled to temporary parental benefit from the government through the Social Insurance Agency (Försäkringskassan), as compensation for salaries lost when staying home from work to care for a sick child. The employment rates in Sweden have been over 75% for both men and women in the working age and more than 80% of children attend day-care centres until the age of five and then compulsory school. TPB can therefore be regarded as a relatively reliable measurement of how much children and their caregivers have to stay home due to the children’s illness. The aim of this study was to investigate whether days of TPB 2 years before and 2 years after tonsil surgery for SDB differ from the expected days of TPB based on data from the entire paediatric population of the region. The hypothesis was that, because of increased morbidity in children with SDB, there would be more days of TPB before surgery and that, after surgery, the number of TPB days would be comparable with the general population.

## Methods

This is a retrospective, cross-sectional study conducted at the Department of Otorhinolaryngology (ORL), Head and Neck Surgery, at Sahlgrenska University Hospital in Gothenburg, Sweden, a tertiary referral hospital serving an underlying population of approximately 1.5 million. Children with SDB due to tonsillar hypertrophy, aged 2–11 years, who underwent tonsil surgery, with or without adenoidectomy, at the Department of ORL in 2014 and 2015 were included. The medical records including the ICD-10 codes EMB10, EMB15, EMB20, EMB30 and EMB99 covering tonsillectomy (TE), tonsillotomy (TT) and adenoidectomy (A) in 2014 and 2015 were reviewed. Data on diagnosis, age, gender, general health and preoperative symptoms were recorded. Both tonsillectomy and tonsillotomy were included in the search, as both surgical methods are used in Sweden to treat paediatric SDB, with a predominance of tonsillotomy. In Sweden, the recommended surgical treatment for SDB is TE or TT + / − A, which explains why patients who underwent isolated adenoidectomy were not included.

Because the objective of this study was to examine TPB data two years prior to surgery and two years after surgery, no children under the age of two were included. Children with SDB who needed in-patient care were not included in the study to avoid heterogeneity associated with specific medical syndromes or severe underlying disease. Children with a protected identity were also excluded. Children who underwent more than one tonsil surgery during the study period were only included once and the date of the first surgery was used as the pivot point.

TPB is available to all Swedish citizens. The insurance is limited to a maximum of 120 days of TPB a year for each child. The TPB is coupled to the child, so, for example, close relatives other than the parents who are employed can also receive TPB if they stay home from work to care for the child. In Sweden, the employment rates for both genders in the age group 16–64 years was 76% and 77%, respectively, in 2014 and 2015. During the same time period, 82% and 83% of children aged 1–5 were enrolled in day-care centres and older children were at school on weekdays. Children were not allowed to attend day care centres or school when they were ill and no medications, such as antibiotics or analgesics, were given at day-care centres or schools. Parents were unable to receive TPB if the other parent was on parental leave. The only situation in which both parents could use TPB at the same time was if the child was admitted to hospital. For reimbursement purposes, TPB could be used as whole days or part of days. In this study, however, all days (whole days or part of days) related to the child were treated as whole days of TPB. The total number of days with TPB was used as a proxy for the number of days the child needed to stay at home due to sickness during the study period. Data from the Swedish Social Insurance Agency on all children aged 2–11 years, registered in 2014 in the region of Västra Götaland, *n* = 36,273, were used to calculate the expected number of days of TPB in relation to age and gender during the observation period.

All Swedish citizens are identified by a unique social security number. Data on TPB for the children undergoing surgery were obtained from the Swedish Social Insurance Agency based on the child’s social security number in the medical records. Data on days of TPB were retrieved two years before and 2 years after the day of surgery for the children undergoing surgery. As tonsil surgery as such generates days with parental benefits during the postoperative care and recovery, TPB during the first 31 days after surgery were excluded from the analyses in a second step.

The study was approved by the Regional Ethics Committee of Gothenburg, number 601–17.

## Statistical analyses

The observed number of days of TPB two years before and two years after surgery was calculated for the 440 children undergoing tonsil surgery. The observed number of days was compared with the expected days of TPB. Age- and sex-specific expected TPB days were calculated for all the children in the same age interval in the region of Västra Götaland with the above 440 excluded (*n* = 36,723). The difference between the observed and expected number of TPB days was calculated and Fisher’s test for pairwise comparisons was used to test the difference between the observed and expected number of days. The results were shown in mean values and standard deviations (SD). Two-sided *p* values were used and *p* < 0.05 was considered to be statistically significant.

## Results

**Fig. 1:**
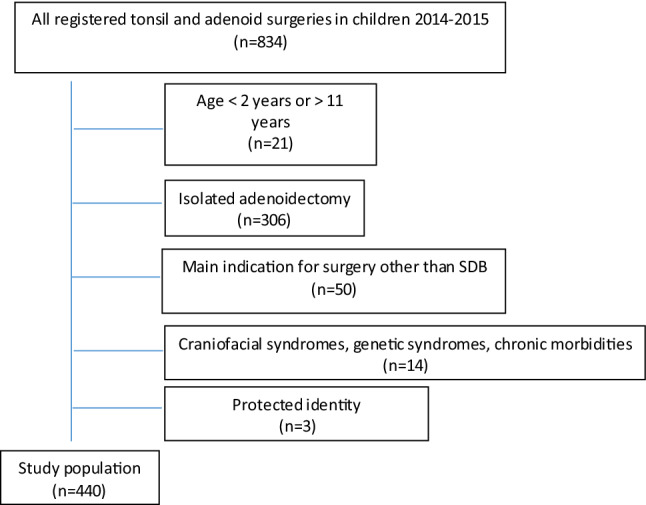
Flow chart of the study population. All children aged 2–11 undergoing surgery at Sahlgrenska University Hospital in 2014 and 2015 with the surgical codes EMB10, EMB15, EMB20, EMB30 and EMB99 were screened. Only children in whom SDB was the main indication for tonsil surgery and that were planned for day surgery were included (*n* = 440). Reasons for exclusion are listed

From the medical records for 2014 and 2015, 834 patients were identified with the designated ICD codes. A total of 440 patients, 177 girls and 263 boys, met all the inclusion criteria and none of the exclusion criteria and were thus included in the study (Fig. 1). All the patients who were planned for day surgery were included, even those who were converted to overnight care on the day of surgery due to complications such as postoperative bleeding or slow recovery (*n* = 49) and a long journey home (*n* = 1).

**Fig. 2:**
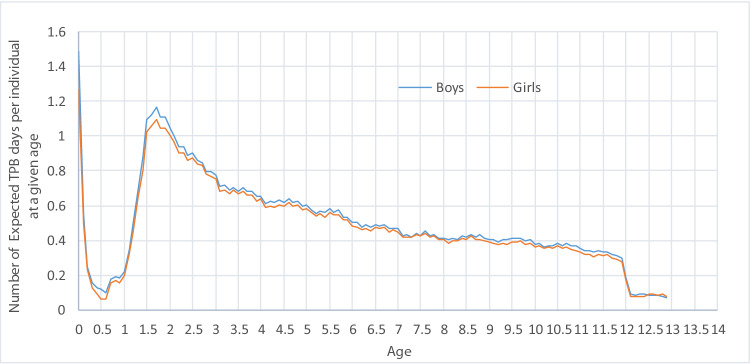
The expected number of days with TPB for each individual age calculated for intervals of 0.1 years (36.5 days). Based on data from the entire population of Västra Götaland, *n* = 36,273

The baseline characteristics for the 440 children are shown in Table 1. The mean age of the children was 4.5 years (2.0–10.6 years). There was no difference in mean age between boys and girls. The most common symptom was snoring (81%), followed by apnea (64%), while the most common associated morbidity was tonsillitis (Table 2). The days of TPB 2 years before and two years after surgery were compared with the days of TPB for all children in the region of the same age and gender (Fig. 2 and Table 3). Of the 36,723 children in the region used to calculate the expected days of TPB, 9% (*n* = 3,251) had no registered days of TPB in the two years before and the 2 years after 2014. In the study group, 43 children undergoing surgery (9.8%) had no reported TPB 2 years before and 2 years after surgery.

**Table 1:** Age, gender and weight of the 440 children with SDB undergoing tonsil surgery with or without adenoidectomy at Sahlgrenska University Hospital in 2014–2015 planned for day surgery

Characteristic	Total (*n* = 440)
Age, year	
Range	2.1–10.6
Mean ± SD	4.5 ± 1.7
Median	4.2
Median, boys	4.3
Median, girls	4.2
Gender	
Male gender, *n* (%)	263 (59.8)
Female gender, *n* (%)	177 (40.2)
Height, cm ± SD	106.3 ± 13.1
Weight, kg ± SD	18.0 ± 6.1
Weight class (IsoBMI)	
Obese, *n* (%)	19 (4.3)
Non-obese, *n* (%)	297 (67.5)
No data, *n* (%)	124 (28.2)

Obesity according to the International Obesity Task Force Classification [19]

**Table 2:** Reported symptoms of SDB and comorbidities among the 440 children with SDB undergoing tonsil surgery with or without adenoidectomy at Sahlgrenska University Hospital in 2014–2015 planned for day surgery

*N* = 440	*n* (%)
Preoperative symptoms	
Snoring	357 (81)
Apnoea	283 (64)
Daytime sleepiness	121 (27)
Mouth breathing	142 (32)
Nasal congestion	121 (27)
Hyponasality	43 (10)
Thick speech	26 (6)
Dysphagia	95 (22)
Poor weight gain	58 (13)
Enuresis	10 (2)
Poor neurocognitive development	10 (2)
Hyperactivity	14 (3)
Related preoperative morbidities	
Recurrent tonsillitis	72 (16)
Recurrent otitis media	55 (13)
Secretory otitis media	88 (20)
Recurrent upper respiratory tract infection	52 (12)
Comorbidities total	88 (20)
Asthma	31 (7)
Allergic rhinitis	5 (1)
Contact allergy	2 (0)
Airway allergy	5 (1)
Food allergy	12 (3)
Epilepsy	1 (0)
ADHD	1 (0)
Heart disease	3 (1)
Other	25 (6)

Related preoperative morbidities were secondary indications for surgery. SDB due tonsillar hypertrophy was the main indication

**Table 3:** Days of parental benefits (TPB) 2 years before and 2 years after tonsil surgery with or without adenoidectomy in children with SDB aged 2–11 undergoing surgery at the Department of ORL, Sahlgrenska University Hospital 2014–2015

	Observed TPB days 2 years before Surgery	Expected TPB days 2 years before surgery	Difference	*p* value	Observed TPB days 2 years after surgery	Expected TPB days 2 years after surgery	Difference	*p* value
	mean ± SD	mean ± SD	mean ± SD		mean ± SD	mean ± SD	mean ± SD	
Boys (*n* = 265)	18.7 ± 22.2	17.5 ± 3.8	1.2 ± 22.1		18.0 ± 18.9	13.6 ± 2.6	4.4 ± 18.9	
Girls (*n* = 175)	19.5 ± 32.3	16.8 ± 3.5	2.7 ± 31.8		18.6 ± 18.1	13.1 ± 2.3	5.5 ± 17.7	
All (*n* = 440)	19.0 ± 26.6	17.3 ± 3.7	1.8 ± 26.4	0.17	18.2 ± 18.5	13.4 ± 2.5	4.8 ± 18.4	< 0.001
All (*n* = 440) with 31 days after surgery excluded	19.0 ± 26.6	17.3 ± 3.7	1.8 ± 26.4	0.17	13.6 ± 16.7	12.8 ± 2.4	0.9 ± 16.5	0.27

The data were also analysed excluding TPB during the first 31 days after surgery. Expected days of TPB were calculated based on all children aged 2–11 in the region of Västra Götaland in 2014 (*n* = 36,273)

The main indication for surgery in all cases was SDB due to tonsil hypertrophy with or without adenoid hypertrophy. Forty children also had concomitant recurrent tonsillitis. Regarding the type of surgery, the most common procedure was TT + A in 369 children (83.9%), followed by TE + A in 44 cases (10%). In all, 16 (3.6%) underwent TT without adenoidectomy and 11 (2.5%) underwent TE without adenoidectomy. Twenty one children experienced a recurrence of tonsil hypertrophy after a previous tonsillotomy. Fifteen of them underwent a tonsil surgery a second time during 2014–2015, (7 TT and 8 TE). Six children underwent two tonsil surgeries during the follow-up period (3 TT + TT and 3 TT + TE).

Thirty-seven (8%) patients experienced postoperative complications during the first month after surgery. Fifteen children had a postoperative infection that required treatment, ten were admitted to hospital due to bleeding, two had bleeding but were not admitted and ten contacted the hospital because of pain.

## Discussion

In this study of children with SDB undergoing day surgery on the tonsils, with and without adenoidectomy, there was no increase in the number of days the parents had to stay home with their child due to sickness, 2 years before and 2 years after surgery, compared with the expected number of days, calculated from data on TPB in a large population of children.

Although children with SDB are known to have associated morbidity and affected quality of life [8, 13], the results do not show that children undergoing surgery for SDB have a greater need to stay at home from day-care centres and schools than other children. To the authors’ knowledge, this has not previously been explored. Many of the known conditions associated with SDB, such as daytime sleepiness, impaired cognition and alertness, may not be enough to cause the child to have to stay home, although they can impose a large negative effect on the child’s quality of life. In a population-based study from Sweden, we have previously shown that the awareness of SDB symptoms is comparatively low among the caregivers of children with moderate to severe symptoms and does not often lead to healthcare contacts [14]. This further highlights the need to be observant of the symptoms and signs of SDB in children and, when present, to make a thorough examination, diagnosis and treatment of SDB in children.

The increased health utilisation seen in children with SDB due to an increased number of upper respiratory tract infections (URI) [8] did not result in the higher utilisation of TPB in this study. The reason could be that URIs are common in all children at a younger age and that many of them would not cause a parent to keep the child home from the day-care centre or school.

Only children undergoing surgery in day surgery were included in the study. As a result, none of the children included in the study had other serious illnesses, syndromes or severe obstructive sleep apnea (OSA), as those children are usually admitted to inpatient care. Had they been included in the study, we would have seen a larger number of TPB days, probably both before and after surgery, which would have affected the results. During the first 2 years after surgery, the SDB group had 4.8 more TPB days than expected, which is statistically significant. As such, however, surgery to the tonsils does generate days of TPB in the recovery period of the child. For this reason, the calculations were also made with the first 31 days after surgery excluded in the follow-up period 2 years after surgery and the difference in the number of TPB compared with the expected number then disappeared. The 4.8 days’ difference during the first month after surgery is likely to be associated with the postoperative recovery of the child and is also comparable to the TPB use related to tonsil surgery found in previous studies [15].

TPB cannot be seen as a completely reliable measurement of morbidity. It does, however, tell us how many days caregivers have had to stay home from work due to their children’s illness and may thus give us an idea of the economic effects of caregivers having to stay home to take care of their sick children. These effects do not appear to be greater in children with SDB compared with the rest of the population.

It is important to highlight the fact that tonsil surgery in SDB has been proven effective in treating SDB symptoms [11, 16]. It has both short- and long-term effects on the well-being and development of the child during a critical time period in life. In Sweden, the predominant surgical technique used in children with SDB is tonsillotomy, which is associated with fewer surgical complications and the faster recovery of the child compared with traditional tonsillectomy [17]. However, tonsillotomy carries a greater risk of tonsillar regrowth and the need for secondary surgery than tonsillectomy, especially in the youngest children [18].

The use of TPB in this study as a proxy for morbidity was founded on the prerequisite that the majority of parents of young children, both men and women, were employed in Sweden during the study period. If they had had to stay at home with a sick child, it is likely they would have used TPB reimbursement for lost salary. Moreover, TPB insurance is available to all parents, as well as other caregivers of the child who are employed (grandparents, etc.). The weakness of using TPB as a proxy for morbidity is that the caregiver decides when to stay at home and no doctor’s examination is required. This could result in misuse and, according to one report from the Swedish Social Insurance Agency, the level of cheating in the TPB system is around 14% (Social Insurance report 2018:1, ISSN 1654–8574) indicating that the problem is minor and should not differ between the groups.

One important strength of this study was that we were able to use data from the entire population of children in the region to calculate the expected days of TPB and that this cohort comprised more than 36,000 children.

One weakness when using TPB in this context is that caregivers may already be at home due to unemployment, their own illness or taking care of another child, but there is no obvious reason why these conditions would differ between the children with SDB and other children.

The TPB data do not enable us accurately to describe what morbidity of the child caused the parent to stay at home. Further research is needed based on primary care data and self-reported symptoms associated with children with SDB staying home from day-care centres and school.

## Conclusion

In this study of children with SDB undergoing day surgery to the tonsils, with and without adenoidectomy, the number of days the parents had to stay home with their children due to sickness, 2 years before and 2 years after surgery, was comparable to the general child population in the same region. Although children with SDB are known to have increased morbidity associated with SDB, the need for caregivers to stay home from work to take care of their children does not appear to be greater.

## Data Availability

The authors own the data.

## References

[1] Lumeng JC, Chervin RD (2008). Epidemiology of pediatric obstructive sleep Apnea. Proceed Am Thor Soc.

[2] Biggs SN, Nixon GM, Horne RS (2014). The conundrum of primary snoring in children: what are we missing in regards to cognitive and behavioural morbidity?. Sleep Med Rev.

[3] Bourke R, Anderson V, Yang JS (2011). Cognitive and academic functions are impaired in children with all severities of sleep-disordered breathing. Sleep Med.

[4] Montgomery-Downs HE, Crabtree VM, Gozal D (2005). Cognition, sleep and respiration in at-risk children treated for obstructive sleep apnoea. Eur Respir J.

[5] Mulvaney SA, Goodwin JL, Morgan WJ (2006). Behavior problems associated with sleep disordered breathing in school-aged children–the Tucson children's assessment of sleep apnea study. J Pediatr Psychol.

[6] Tan HL, Alonso Alvarez ML, Tsaoussoglou M (2017). When and why to treat the child who snores?. Pediatr Pulmonol.

[7] Castro-Rodriguez JA, Brockmann PE, Marcus CL (2017). Relation between asthma and sleep disordered breathing in children: is the association causal?. Paediatr Respir Rev.

[8] Tarasiuk A, Greenberg-Dotan S, Simon-Tuval T (2007). Elevated morbidity and health care use in children with obstructive sleep apnea syndrome. Am J Respir Crit Care Med.

[9] Tarasiuk A, Simon T, Tal A (2004). Adenotonsillectomy in children with obstructive sleep apnea syndrome reduces health care utilization. Pediatrics.

[10] Friedman M, Tanyeri H, La Rosa M (1999). Clinical predictors of obstructive sleep apnea. Laryngoscope.

[11] Marcus CL, Moore RH, Rosen CL (2013). A randomized trial of adenotonsillectomy for childhood sleep apnea. New England J Med.

[12] Gudnadottir G, Ellegard E, Hellgren J (2018). Intranasal budesonide and quality of life in pediatric sleep-disordered breathing: a randomized controlled trial. Otolaryngol Head Neck Surg.

[13] Jackman AR, Biggs SN, Walter LM (2013). Sleep disordered breathing in early childhood: quality of life for children and families. Sleep.

[14] Gudnadottir G, Ehnhage A, Bende M (2016). Healthcare provider contact for children with symptoms of sleep-disordered breathing: a population survey. J Laryngol Otol.

[15] Gudnadottir G, Tennvall GR, Stalfors J (2017). Indirect costs related to caregivers’ absence from work after paediatric tonsil surgery. Eur Arch Otorhinolaryngol.

[16] Mitchell RB, Kelly J (2007). Outcomes and quality of life following adenotonsillectomy for sleep-disordered breathing in children. ORL J Otorhinolaryngol Relat Spec.

[17] Borgstrom A, Nerfeldt P, Friberg D (2017). Adenotonsillotomy versus adenotonsillectomy in pediatric obstructive sleep apnea: an RCT. Pediatrics.

[18] Odhagen E, Sunnergren O, Hemlin C (2016). Risk of reoperation after tonsillotomy versus tonsillectomy: a population-based cohort study. Eur Arch Otorhinolaryngol.

[19] Cole TJ, Bellizzi MC, Flegal KM (2000). Establishing a standard definition for child overweight and obesity worldwide: international survey. BMJ.

